# Socioeconomic Inequality in the Use of Long-Term Care among European Older Adults: An Empirical Approach Using the SHARE Survey

**DOI:** 10.3390/ijerph18010020

**Published:** 2020-12-22

**Authors:** Javier Lera, Marta Pascual-Sáez, David Cantarero-Prieto

**Affiliations:** Department of Economics & Group of Health Economics and Health Service Management, University of Cantabria—IDIVAL, Avenue Los Castros s/n, 39005 Santander, Spain; Marta.Pascual@unican.es (M.P.-S.); David.Cantarero@unican.es (D.C.-P.)

**Keywords:** aging, inequality in use, inequality, long-term care, SHARE

## Abstract

The increase in the proportion of elderly people in developed societies has several consequences, such as the rise in demand for long-term care (LTC). Due to cost, inequalities may arise and punish low-income households. Our objective is to examine socioeconomic inequalities in LTC utilization in Europe. We use the last wave from the Survey of Health, Aging, and Retirement in Europe SHARE (Munich Center for the Economics of Ageing, Munich, Germany), dated 2017, to analyze the impact of socioeconomic status (SES) on LTC. For this purpose, we construct logistic models and control for socioeconomic/household characteristics, health status, and region. Then, concentration indices are calculated to assess the distribution of LTC. Moreover, we also analyze horizontal inequity by using the indirect need-standardization process. We use two measures of SES (household net total income and household net wealth) to obtain robust results. Our findings demonstrate that informal care is concentrated among low-SES households, whereas formal care is concentrated in high-SES households. The results for horizontal concentration indices show a pro-rich distribution in both formal and informal LTC. We add new empirical evidence by showing the dawning of deep social inequalities in LTC utilization. Policymakers should implement policies focused on people who need care to tackle socioeconomic inequalities in LTC.

## 1. Introduction

One consequence of the improvement in life expectancy rates is an increase in the number of people suffering from degenerative conditions or memory impairments due in the next decades [1,2]. Elderly people may need care all day long, and getting professional carers or even a bed in nursing homes is unaffordable for many patients due to the high associated cost. According to the European Institute for Gender Equality (EIGE) [3], 52% of European households cannot afford long-term care (LTC). Therefore, responsibility for care falls upon non-professionals, mainly women [4]. EIGE [5] estimates that 62% of the people who provide care are women. This affects their careers, social relationships, and well-being [6,7,8].

Moreover, the economic and political crisis of the late 2000s has deeply affected European economies, and their governments have implemented general restrictive budgetary policies to reduce their high public debt rates [9]. These policies included the tightening of long-term care (LTC) eligibility criteria and cuts in public funds [8].

Thus, important inequalities and inequities may arise in LTC utilization among elderly people [1]. Some studies point out the pro-rich distribution formal LTC services have [10,11,12]. In this regard, Jones [13] concluded that social class matters in the diagnosis and care of dementia. Moreover, there is a loss in social status caused by the diagnosis of this condition according to this author. Lindgren et al. [14] proved the existence of a significant gap in equity in dementia care between nationals and immigrants in Sweden. Factors explaining LTC use may vary with time. Differences in LTC utilization tend to disappear some years before death. Being single and having a low income are related to a higher probability of being a formal care user [15]. Chuakhamfoo et al. [16] focused on patients suffering from dementia in low-income rural areas. Their study found that 80% of the care provided was informal. According to Hu et al. [17], in China, people of low socioeconomic status need more LTC. Recent evidence also proves the importance of the offer of formal care from institutions. Floridi et al. [18] demonstrated that low-income households tend to mix more formal and informal care than their high-income peers. These authors concluded that LTC defamilization (offering families the possibility of institutional care for their relatives) may reduce LTC inequalities in later life.

European institutions are worried about the future of LTC. According to a European Commission report [8], European countries are facing similar challenges. Firstly, the insufficient development of public formal LTC reduces its access and adequacy. Moreover, there is no complementarity between informal and formal LTC. For example, being an informal care user should not be a reason for not being a formal care user. Governments could take advantage of the high informal care development to better protect those individuals with greater needs by mixing both types of care. Thus, tradeoffs between both types of care have not been enhanced. Secondly, the volume of LTC demanded will grow, leading to a reduction in the quality of LTC services if new measures are not implemented. Thirdly, regarding employment, mostly women are in charge of care, which in most cases is not provided in a formal way. Finally, the European Commission is concerned about the financial sustainability of the LTC system. Although European countries must face similar challenges, regional disparities remain important. While some countries focus on providing services, others focus on cash benefits. Moreover, formal services are less developed in some Eastern and Southern European countries [8].

To tackle these challenges, the European Commission has made some policy recommendations. First, formal home care should be developed without reducing the supply of residential institutions. Second, there should be greater control of cash benefits. Users must provide evidence that public funds are used for LTC. Third, informal carers should be supported by training them, giving them more social security rights, or supporting their labor participation.

Other studies have already studied the inequalities and inequities in the use of LTC. The report by Rodrigues, Ilinca, and Schmidt [19] analyzed the equity in LTC use in Europe. To do so, they used data from the second wave of the SHARE survey dated 2006−2007. They focused on home care services and informal care. Authors developed concentration curves, concentration indices, and horizontal indices. They conclude that, in general, the main drivers for differences in the use of formal LTC are factors related to need. On the other hand, they found that socioeconomic status matters in the use of informal care although they controlled for need (horizontal inequity). The same authors developed a similar study in 2017 [20] by using data from the fifth wave of the SHARE survey. Again, they analyzed home care services as formal care and calculated horizontal concentration indices. In general, they found pro-poor distributions in home care. Nevertheless, some inequities started to arise in some countries such as Spain. After controlling for needs, home care is more used by rich households.

Hence, this paper focuses on the first concern by assessing inequalities due to socioeconomic status (SES) in LTC utilization in ten European countries. The aim is to analyze whether SES affects LTC service utilization in later life. Considering the past studies [19,20], we update their evidence and analyze the evolution of socioeconomic inequalities in LTC.

The structure of this paper is as follows. Section 2 presents the methodology and data. Section 3 is focused on empirical analysis. Section 4 discusses our results, and Section 5 presents the conclusions and policy implications.

## 2. Materials and Methods

The Survey of Health, Aging, and Retirement in Europe (SHARE) was our data source. The SHARE survey is a cross-national panel database focused on people aged 50 or older that covers 28 countries. It is a multidisciplinary survey that includes microdata on personal characteristics, socioeconomic status, health, and personal networks. We used the last data available, which were from Wave 7 (2017).

**Figure 1:**
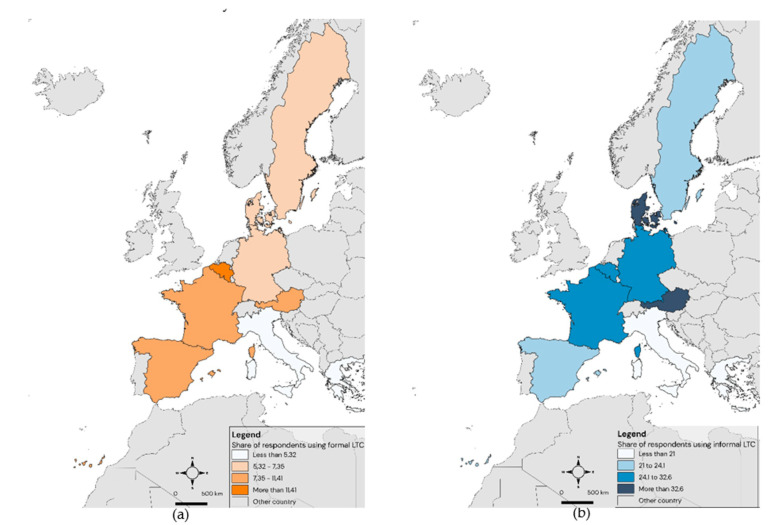
Share of respondents that declare using formal care (**a**) and informal care (**b**). Source: authors’ elaboration based on the Survey of Health, Aging, and Retirement in Europe (SHARE) (Wave 7).

Our variables of interest are FormalCare (nursing home admissions and professional care received at home) and InformalCare (whether a non-professional from the respondent household or from outside it provides care). Figure 1 represents formal and informal LTC utilization according to Wave 7 of the SHARE survey.

Variables capturing personal characteristics include age, gender, and marital status. We created three age groups, as other studies have [21,22]: people aged between 50 and 65 years old, those aged 66–80, and those who are older than 80. A covariate capturing whether the respondent is female or not (i.e., male) was considered due to the relevance of gender [23]. Household (HH) composition may affect the respondent’s probability of receiving LTC [24,25]. Thus, variables capturing respondents’ marital status (Single), the household size (HHsize), the number of descendants, and whether they live near or in the respondents’ household were included (NChild and ChHH). We also decided to include two variables to capture the effects of the number of people living in the same household as the respondent (hhsize) and where the respondent lives, whether it is a rural or urban area (urban).

Moreover, health status is a key determinant behind healthcare utilization, particularly in LTC services. The following variables were introduced: self-assessed good health status (SAGHS), the number of limitations in activities of daily living (ADLs), and the number of chronic diseases (NCD). We also included three dichotomous variables capturing the geographical area of the respondent’s living place. These areas were constructed following the report by Zigante [26] and the European Commission Thesaurus criteria [27]. Northern Europe is composed of Denmark and Sweden. Southern Europe comprises Greece, Italy, and Spain. Western Europe comprises Austria, Belgium, France, and Germany. The last area is Eastern and Central Europe, which is composed of the Czech Republic.

Table 1 gives a description of the variables and a summary of their main statistics. In addition, Table 2 differentiates the descriptive statistics by country: Austria (AT), Germany (DE), Sweden (SE), Spain (ES), Italy (IT), France (FR), Denmark (DK), Greece (GR), Belgium (BE), and the Czech Republic (CZ).

Our variables of interest are binary: FormalCare and InformalCare. They take the value 1 if the respondent receives formal care or informal care (with probability p) and 0 if not (with probability (1 − p)). The probability of receiving LTC (p) is a function of two vectors: one of explanatory variables (x) and the other of unknown parameters (β). Thus, the discrete choice models are as follows:

Prob(h = 1) = G(x, β),(1)

Prob(h = 0) = 1 − G(x, β).(2)

Considering this, the latent interpretation from both equations leads to the following specification:

hi = 1 if hi* > 0,(3)

hi = 0 if hi* ≤ 0,(4)

where

h* = x′β + ε.(5)

Therefore, the election between a probit and a logit model depends on the assumptions made on ε. If its cumulative distribution is the logistic one, then logit models must be used. Moreover, Maddala [28] argues that the logit models perform better since they are less sensitive to uneven sample frequency problems. Other studies have used the logistic model to study LTC [29,30,31,32]. Then, we constructed a logistic regression model to estimate the impact of the different factors on LTC utilization. In the logit model, the conditional probability allows the predicted probabilities to be bounded between 0 and 1 by assuming that the conditional probability takes the following form:

*p* = Prob(y = 1│X) = exp((X′β)/(1 − exp(X′β)) (6)

Considering that the non-linearity of our models prevents us from interpreting the coefficients as usual, the odds ratios were calculated. These are the ratios of the probability of success and the probability of failure:

ln(*p*/(1 − p)) = X′β (7)

Moreover, it is necessary to remember that Western Europe and the primary education group are considered the reference categories. Considering the existent literature concerning healthcare inequalities, we used the concentration index (CI) proposed by the existing literature [33,34,35]. CI has been used in several studies, such as that by Ilinca et al. [20], where the factors driving inequality and inequity in home care are analyzed.

CI = (2/µ) × cov(hi, ri)(8)

Here, hi and µ are the variables related to LTC utilization and its average, respectively, and ri is the relative rank of the individual i in the socioeconomic distribution. We use household net total income and household net wealth to provide robust results. Recent literature [36] proved the impact on the final results of the measurement chosen. CI is bounded between −1 and +1. If CI takes a positive value, it means that the distribution of LTC favors high-socioeconomic-status individuals. Thus, a CI below zero would mean that LTC is concentrated in the poorest households. Our LTC variable is binary, so the CI would be bounded between 0 and 1, and depending on the mean of the LTC variable, CI could tend to zero, which would bias our results [29]. CI thus needs to be transformed [37]:

CCI = 4 × µ × CI = 8 cov (hi, ri).(9)

Finally, we present horizontal inequity indices (HIs) in the use of formal and informal care. These horizontal indices help us to analyze the effect of SES on the LTC use by controlling for need factors and non-need factors associated with LTC utilization. In other words, we can assess whether SES plays an important role although respondents have serious impairments and need LTC. We considered two set of variables: need (N) LTC determinants (age, gender, self-assessed health status, the number of limitations in activities of daily living, and the number of chronic diseases) and non-need (Z) LTC determinants (marital status, education level, household composition, number of descendants, and the region in which the respondent lives) [37,38,39,40]. We followed the indirect need-standardization process [35]. First, we divide the factors explaining LTC utilization (hi) into need and non-need factors:

(10) $$h_{i}\;=\;\alpha +\sum_{k}\beta_{k}N_{\mathrm{ik}}+\sum_{j}\delta_{j}Z_{\mathrm{ij}}{+\varepsilon }_{i}$$

We estimate this model and keep the predicted values $\hat{h_{i}}$. Then, we can calculate the indirect standardized values $\left(h_{i}^{\prime }\right)$ by subtracting the CI for the need-predicted LTC use from the CI corresponding to the real use and summing the mean [41,42]:

(11) $${h^{\prime }}_{i}={\;h}_{i}\;-\hat{h_{i}}+\;\bar{h}$$

Then, the concentration indices for the need-predicted use can be calculated in a similar way as before. The results yield horizontal concentration indices. The interpretation is similar to that for the CI: values below zero indicate a pro-poor distribution, whereas positive values indicate pro-rich inequity.

## 3. Results

Table 3 shows the results for all the logistic models. Similar effects for both formal and informal LTC were demonstrated for variables regarding age, gender, marital, and health status. LTC utilization increases with age, the fact of being single and a woman, the number of limitations in daily living activities, and the number of chronic diseases. On the other hand, with at least a good health status and a greater number of household members, LTC utilization is reduced.

Differences arise when analyzing the education level, whether the household is in a rural or urban area, and the residence region. The higher the education level received, the higher the probability of receiving formal LTC. On the other hand, a low education level might be associated with higher informal care use. This supports the idea that formal LTC is more common among high-education households. Moreover, respondents living in an urban area are more prone to being formal care users, whereas those living in rural areas tend to use more informal LTC. Regarding the regions, all of them have less-developed formal LTC services in comparison to Western Europe. In the case of informal care, Eastern and Northern Europe respondents tend to use more informal care than their Western peers.

Table 4 includes the CIs for both formal and informal care by country. Overall, our results suggest that most of the countries analysed follow the same pattern. Formal care is, in general, concentrated in high-SES households. Czech Republic and Greece are the only countries where pro-poor formal LTC can be found. In the case of the CZ, it is only significant when the net household wealth is used as an indicator of SES. Moreover, informal care is generally concentrated in low-SES households. In this case, all the CIs are statistically significant using both SES indicators. In general, the pro-rich or pro-poor distribution remains the same although the rank variable changes. Nevertheless, when the household wealth is used as the rank variable. The pro-rich inequality is deeper. For informal care, there is not a similar effect of the rank variable. In some countries, the household wealth increases the pro-poor distribution, while in other countries this inequality became less deep.

We also analyzed the horizontal inequity in LTC services (Table 5). To do so, we calculated the HIs for both formal and informal LTC. All the horizontal indices are positive regardless the rank variable chosen. As in the concentration indices, the election of the rank variable does not have a similar effect in the distribution in the countries. In some countries, household wealth increases the pro-rich inequity and in other countries inequities are smaller. This shows a pro-rich distribution in the use of LTC. As a conclusion, both formal and informal care are concentrated in high-SES households, which show deep horizontal inequities in the LTC use.

A clear difference can be seen in the results for inequality and inequity. Inequality just measures the differences in LTC use by SES. On the other hand, horizontal inequity studies the role of SES taking into consideration the need for LTC. This is whether for the same level of care needs due to health status, the SES plays a role in the decision of using LTC. The positive values for horizontal concentration indices, mean that two individuals with the same level of care needs, have different LTC use depending on their SES. We could conclude that respondents with higher care needs suffer a greater impact of their SES on their LTC use.

## 4. Discussion

In this paper, we examined socioeconomic inequalities and inequities in the use of formal and informal LTC. This investigation focused on European older adults from ten countries. We developed CIs and HIs using two approaches for SES: the net household income and the net household wealth. Our results suggest a pro-poor distribution for informal care and a pro-rich distribution for formal care when analyzing LTC-access inequalities. On the other hand, we found pro-rich distributions when concentration indices were calculated for horizontal inequity. This means that LTC is concentrated among high-SES households.

Our results regarding those variables capturing personal characteristics, socioeconomic status, and health status are consistent with those of previous studies [10,20,43]. We also demonstrated the important role that family networks play in determining LTC service utilization. People with descendants receive more support and informal care than do those who declare being childless or those whose descendants do not live near them. Moreover, we found a similar relationship between being single and receiving LTC in both cases. Single people use more LTC services than their non-single peers [10,44,45,46]. The composition of the variable InformalCare may determine the fact that single people receive informal care. Two items compose the variable: receiving care from someone inside or outside the household. In this regard, Schmidt [36] proved the existence of differences in the use of home care between single and non-single people depending on their socioeconomic status.

Regarding the education level, we found different effects on informal and formal care according to our results. Respondents with a high education level are more prone to using formal care, while informal care is more widespread among low-education respondents. These results are consistent with our CIs. Both effects may be caused by the lack of information and by the impossibility of affording the high cost of a professional carer [11,12,47,48]. Our results suggest that SES inequalities are higher in formal LTC utilization than in informal care. These inequalities may have increased in the last years due to the restrictive policies implemented on LTC programs and their funds.

Moreover, we found pro-rich distributions when concentration indices were calculated to analyze horizontal inequity. Our results can be compared with other similar studies developed by Rodrigues et al. [19] and Ilinca et al. [20]. The first study developed in 2014 did not find any significant horizontal inequities in LTC. The second one [20] used data from 2013 and found little evidence of significant horizontal inequities in home care for some European countries. In this regard, our results suggest the existence of a new European pattern of raising new horizontal inequities in LTC. We can conclude that both formal and informal care are concentrated in high-class households when we take into consideration the need for care. These inequities may be partially explained by the fact that people who need others to have information on LTC or their entitlements are concentrated in low-SES households [48]. It would be interesting to have precise data and sufficient observations to deeply analyze these inequities by other characteristics such as illness type.

Finally, we would like to acknowledge some limitations of this study: Firstly, we relied on self-reported data, and the restrictions of this kind of data are well known. Secondly, information of regions inside the countries was not included due to the lack of precise indicators. Thirdly, we compared access to LTC across European countries where the LTC provision and laws are different. Finally, although institutionalized people are always considered eligible in the longitudinal sample, in some countries these people are not eligible. The eligibility depends on national regulations [49]. These limitations can be also understood as future research directions.

## 5. Conclusions

Our paper contributes to the existing literature on equity in LTC. It has several implications for the successful implementation of public policies. Those households and individuals who need more care and/or have low SES are supposed to be well covered by the protective measures developed by public institutions. Nevertheless, we proved growing inequalities in LTC use: while formal care is more widespread among those with high SES, informal care is more used by those with low SES. Moreover, when we analyzed LTC use by household needs, we found that all types of care are concentrated in high-SES households. This proves the existence of two barriers to LTC access: The first is belonging to a low-SES group. The second is that having more disabling conditions makes individuals more prone to having unmet care necessities.

A better European strategy focused on tackling inequalities and inequities in access to LTC is needed. We also recommend that governments promote policies improving their LTC systems as a measure to reduce burdens in access to LTC, especially for those people who need care.

## Figures and Tables

**Table 1:** Variable definitions and summary of statistics.

Variable	Definition	Coding	Mean	SD
Long-Term Care				
InformalCare	Non-professional help inside or outside the household	1: if respondent has received non-professional help inside or outside the household; 0: otherwise	0.2832	0.4506
FormalCare	Professional help at home or institutionalization	1: if respondent has been institutionalized or has received professional help at home; 0: otherwise	0.0913	0.2881
Personal Characteristics				
Female	Gender of respondent	1: if respondent’s gender is female; 0: otherwise (male)	0.5605	0.4963
Age	Age of respondent	Age in years	69.6823	9.7148
Single	Marital status	1: never married, divorced, or widowed; 0: married, living with spouse or not, or registered partnership	0.2933	0.4553
Education	Education level	1: primary education; 2: secondary education; 3: tertiary education	1.8250	0.7916
NChild	Number of descendants that are still alive	Number of children that are still alive	2.1526	1.2767
Household Characteristics				
ChHH	Descendants’ living place	1: If at least one child lives in the same household or the same building as respondent	0.0041	0.0642
HHsize	Household size	Number of people living in the same household as the respondent	2.0274	0.8721
Urban	Respondents’ living area	1: if respondent lives in an urban area; 0: otherwise	0.6771	0.4676
Health Status				
SAGHS	Self-assessed good health status	1: if respondent’s self-assessed good health status is excellent, very good, good, or fair; 0: otherwise (poor)	0.6321	0.4822
ADL	Number of limitations in activities of daily living (ADLs)	Number of limitations in ADLs	0.2792	0.9573
NCD	Number of chronic diseases	Number of chronic diseases	1.9179	1.6332

**Table 3:** Estimates for formal care and informal care.

	Formal Care		Informal Care	
Variable	Coef.	Odds Ratio	Coef.	Odds Ratio
Personal Characteristics				
Female	0.3556 ***	1.4270***	0.1415 ***	1.1520 ***
	0.0478	0.0682	0.0486	0.0560
Age 66 to 80 years	0.5817 ***	1.7890 ***	0.0423	1.0432
	0.0601	0.1075	0.0628	0.0655
Age 80 plus years	1.7870 ***	5.9716 ***	0.7107 ***	2.0354 ***
	0.0656	0.3917	0.0761	0.1548
Single	0.6815 ***	1.9768 ***	0.5967 ***	1.8161 ***
	0.0605	0.1196	0.0666	0.1209
Secondary Education	−0.0919 *	0.9122 *	0.0244	1.0247
	0.0531	0.0484	0.0566	0.0580
Tertiary Education	0.1363 **	1.1460 **	−0.0372	0.9635
	0.0598	0.0686	0.0650	0.0627
NChild	0.0028 ***	1.0028 ***	0.0836 ***	1.0872 ***
	0.0004	0.0004	0.0183	0.0199
Household Characteristics				
ChHH	−0.2824	0.7540	−0.3232	0.7238
	0.3944	0.2974	0.2542	0.1840
HHsize	−0.1669 ***	0.8463 ***	−0.1995 ***	0.8192 ***
	0.0418	0.0354	0.0464	0.0380
Urban	0.2344 ***	1.2642 ***	−0.2925 ***	0.7464 ***
	0.0471	0.0596	0.0504	0.0376
Health Status				
SAGHS	−0.7566 ***	0.4693 ***	−0.5225 ***	0.5931 ***
	0.0500	0.0235	0.0528	0.0313
ADL	0.4994 ***	1.6477 ***	0.4329 ***	1.5417 ***
	0.0184	0.0303	0.0328	0.0506
NCD	0.1347 ***	1.1441 ***	0.1663 ***	1.1810 ***
	0.0134	0.0154	0.0160	0.0189
Country				
Southern Europe	−1.2364 ***	0.2904 ***	−0.5970 ***	0.5505 ***
	0.0623	0.0181	0.0598	0.0329
Eastern Europe	−0.6313 ***	0.5319 ***	0.2249 ***	1.2521 ***
	0.0597	0.0318	0.0637	0.0797
Northern Europe	−0.8903 ***	0.4105 ***	0.7492 ***	2.1154 ***
	0.0797	0.0327	0.0870	0.1841
Constant	−2.8983 ***	0.0551 ***	−1.1377 ***	0.3206 ***
	0.1330	0.0073	0.1479	0.0474
Log pseudolikelihood	−8031.1996		−5767.0237	
Number of observations	35,718		11,629	

Note: Standard deviations are given under the estimates. ***, **, *, denote significance at levels 1%, 5%, and 10%, respectively; Reference categories: Western Europe and primary education. Northern Europe: Denmark and Sweden. Southern Europe: Greece, Italy, and Spain. Western Europe: Austria, Belgium, France, and Germany. Eastern and Central Europe: Czech Republic.

**Table 4:** Inequality in the use of long-term care (LTC).

Ranking Variable	AT	DE	SE	ES	IT	FR	DK	GR	BE	CZ
Informal Care										
HHTotal Income	−0.1875 ***	−0.2038 ***	−0.1634 ***	−0.1254 ***	−0.0936 ***	−0.2159 ***	−0.1672 ***	−0.1250 ***	−0.2164 ***	−0.1689 ***
	(0.0510)	(0.0361)	(0.0309)	(0.0262)	(0.0221)	(0.0309)	(0.0310)	(0.0205)	(0.0262)	(0.0375)
HH wealth	−0.1499 ***	−0.1674 ***	−0.0967 ***	−0.1332 ***	−0.1160 ***	−0.1331 ***	−0.1475 ***	−0.2045 ***	−0.2367 ***	−0.1843 ***
	(0.0526)	(0.0364)	(0.0304)	(0.0261)	(0.0234)	(0.0313)	(0.0308)	(0.0214)	(0.0256)	(0.0371)
Formal Care										
HH Total Income	0.0176 *	0.0222 ***	0.0269 ***	−0.0026	0.0154 **	0.0003	0.0070	−0.0361 ***	0.0275 **	−0.0103
	(0.0089)	(0.0085)	(0.0101)	(0.0089)	(0.0072)	(0.0112)	(0.0089)	(0.0087)	(0.0116)	(0.0065)
HH wealth	0.0209 *	0.0375 ***	0.0506 ***	0.0427 ***	0.0243 ***	0.0111	0.0161	−0.0306 ***	0.0550 ***	−0.0175 ***
	(0.0086)	(0.0082)	(0.0097)	(0.0086)	(0.0070)	(0.0107)	(0.0087)	(0.0074)	(0.0113)	(0.0057)

Source: authors’ elaboration; Note: ***, **, *, are the significance at level 1, 5 and 10% respectively.

**Table 5:** Horizontal inequity in the use of LTC.

Ranking Variable	AT	DE	SE	ES	IT	FR	DK	GR	BE	CZ
Informal Care										
HH Total Income	0.0880 ***	0.0775 ***	0.1221 ***	0.0944 ***	0.0487 ***	0.0914 ***	0.1001 ***	0.0487 ***	0.0901 ***	0.0761 ***
	(0.0128)	(0.0084)	(0.0074)	(0.0087)	(0.0068)	(0.0076)	(0.0069)	(0.0059)	(0.0063)	(0.0091)
HH wealth	0.0994 ***	0.0681 ***	0.1018 ***	0.0732 ***	0.0539 ***	0.0789 ***	0.0797 ***	0.0585 ***	0.0727 ***	0.1024 ***
	(0.0135)	(0.0091)	(0.0080)	(0.0083)	(0.0068)	(0.0074)	(0.0071)	(0.0058)	(0.0063)	(0.0093)
Formal Care										
HH Total Income	0.1277 ***	0.0923 ***	0.1363 ***	0.1051 ***	0.0616 ***	0.1081 ***	0.1463 ***	0.0660 ***	0.1167 ***	0.1044 ***
	(0.0140)	(0.0090)	(0.0073)	(0.0105)	(0.0082)	(0.0081)	(0.0066)	(0.0075)	(0.0072)	(0.0102)
HH wealth	0.1360 ***	0.0899 ***	0.1017 ***	0.0918 ***	0.0721 ***	0.0935 ***	0.0982 ***	0.0972 ***	0.1034 ***	0.1150 ***
	(0.0154)	(0.0093)	(0.0079)	(0.0101)	(0.0083)	(0.0083)	(0.0070)	(0.0073)	(0.0072)	(0.0105)

Source: authors’ elaboration; Note: *** is the significance at level 1%.

**Table 2:** Summary of statistics by country.

	AT	DE	SE	ES	IT	FR	DK	GR	BE	CZ
Informal care (%)	41.0148	32.6139	24.1088	22.9379	20.2936	29.6752	38.0952	20.9694	27.5995	45.1754
	(49.2381)	(46.9080)	(42.7945)	(42.0599)	(40.2313)	(45.7027)	(48.5811)	(40.7198)	(44.7158)	(49.7940)
Formal care (%)	10.7383	7.3471	7.3350	9.0217	5.3239	11.4109	6.7636	4.8249	18.9184	7.1810
	(30.9649)	(26.0942)	(26.0753)	((28.6524)	(22.4534)	(31.7992)	(25.1160)	(21.4326)	(39.1696)	(25.8204)
Female (%)	59.0210	52.9102	53.8172	55.6886	54.7803	57.9745	54.1356	57.3544	55.3472	59.9237
	(49.1872)	(49.9218)	(49.8619)	(49.6807)	(49.7765)	(49.3675)	(49.8364)	(49.4643)	(49.7184)	(49.0112)
Age (in years)	70.7449	68.1372	72.1986	71.5682	69.1585	69.6413	67.1915	69.6703	68.3192	70.3550
	(9.2835)	(9.3417)	(8.8213)	(10.2876)	(9.6743)	(10.2909)	(9.6754)	(9.3476)	(10.2364)	(8.5431)
Single (%)	36.3665	24.5194	28.7465	27.6305	22.9411	34.8696	26.1194	28.5620	32.0008	33.1345
	(48.1129)	(43.0259)	(45.2651)	(44.7217)	(42.0500)	(47.6630)	(43.9354)	(45.1784)	(46.6527)	(47.0753)
Primary education (%)	23.3448	11.3247	33.8674	78.5714	68.8231	38.6598	16.6356	52.0237	36.5135	38.0248
	(42.3092)	(31.6937)	(47.3333)	(41.0370)	(46.3267)	(48.7044)	(37.2458)	(49.9673)	(48.1518)	(48.5506)
Secondary education (%)	49.6392	56.4656	33.2077	10.5646	23.1398	37.0831	37.8731	29.8124	27.6942	47.6861
	(50.0065)	(49.5867)	(47.1032)	(30.7417)	(42.1773)	(48.3101)	(48.5147)	(45.7510)	(44.7534)	(49.9524)
Tertiary education (%)	27.0160	32.2096	32.9249	10.8640	8.0371	24.2571	45.4913	18.1639	35.7923	14.2891
	(44.4112)	(46.7341)	(47.0014)	(31.1220)	(27.1897)	(42.8703)	(49.8040)	(38.5610)	(47.9439)	(35.0004)
Number of descendants	2.1064	1.9920	2.2164	2.5501	2.0571	2.3302	2.2384	1.8643	2.1507	2.1155
	(1.4066)	(1.2252)	(1.2165)	(1.5972)	(1.2274)	(1.4200)	(1.2377)	(0.9566)	(1.3817)	(0.9270)
Living with descendants (%)	0.2196	0.3687	0.1885	0.5560	0.8170	0.2426	0.4353	0.3620	0.4739	0.2624
	(4.6822)	(6.0618)	(4.3383)	(7.4368)	(9.0026)	(4.9199)	(6.5846)	(6.0064)	(6.8687)	(5.1164)
Household size	1.8817	1.9573	1.7908	2.2397	2.3449	1.8924	1.8955	2.1316	1.9825	1.9854
	(0.8651)	(0.7202)	(0.5652)	(0.9741)	(0.9966)	(0.8111)	(0.6819)	(0.9239)	(0.8694)	(0.9206)
Living in urban areas (%)	50.7085	59.1251	62.1395	84.6242	65.7284	51.8461	75.4119	84.3353	67.1958	71.1117
	(50.0034)	(49.1669)	(48.5121)	(36.0758)	(47.4673)	(49.9737)	(43.0677)	(36.3528)	(46.9550)	(45.3302)
At least good self-assessed health (%)	61.8136	56.9660	68.4889	55.1112	55.7960	62.4924	73.6629	66.8641	67.8137	67.5573
	(48.5920)	(49.5189)	(46.4633)	(49.7434)	(49.6684)	(48.4216)	(44.0530)	(47.0779)	(46.7239)	(46.8216)
Number of ADL limitations	0.2949	0.2499	0.1976	0.4134	0.2826	0.2674	0.1785	0.1612	0.3313	0.3137
	(1.0098)	(0.8489)	(0.7667)	(1.2438)	(1.0316)	(0.9070)	(0.7494)	(0.7394)	(0.9770)	(0.9620)
Number of chronic illnesses	1.9206	2.0716	1.6510	2.0079	1.6410	1.8451	1.5917	1.9197	2.0387	2.3447
	(1.6258)	(1.7347)	(1.4654)	(1.6905)	(1.5338)	(1.5316)	(1.4685)	(1.5968)	(1.6582)	(1.7571)

Source: authors’ elaboration.

## Data Availability

Restrictions apply to the availability of these data. Data was obtained from Munich Center for the Economics of Aging (MEA) and are available at http://www.share-project.org/data-access.html with the permission of MEA.
